# Modeling heterogeneous (co)variances from adjacent-SNP groups improves genomic prediction for milk protein composition traits

**DOI:** 10.1186/s12711-017-0364-8

**Published:** 2017-12-05

**Authors:** Grum Gebreyesus, Mogens S. Lund, Bart Buitenhuis, Henk Bovenhuis, Nina A. Poulsen, Luc G. Janss

**Affiliations:** 1 Department of Molecular Biology and Genetics, Center for Quantitative Genetics and Genomics, Aarhus University, Blichers Allé 20, P.O. Box 50, 8830 Tjele, Denmark; 2 Animal Breeding and Genomics Centre, Wageningen University, PO Box 338, 6700 AH Wageningen, The Netherlands; 3 Department of Food Science, Aarhus University, Blichers Allé 20, P.O. Box 50, 8830 Tjele, Denmark

## Abstract

**Background:**

Accurate genomic prediction requires a large reference population, which is problematic for traits that are expensive to measure. Traits related to milk protein composition are not routinely recorded due to costly procedures and are considered to be controlled by a few quantitative trait loci of large effect. The amount of variation explained may vary between regions leading to heterogeneous (co)variance patterns across the genome. Genomic prediction models that can efficiently take such heterogeneity of (co)variances into account can result in improved prediction reliability. In this study, we developed and implemented novel univariate and bivariate Bayesian prediction models, based on estimates of heterogeneous (co)variances for genome segments (BayesAS). Available data consisted of milk protein composition traits measured on cows and de-regressed proofs of total protein yield derived for bulls. Single-nucleotide polymorphisms (SNPs), from 50K SNP arrays, were grouped into non-overlapping genome segments. A segment was defined as one SNP, or a group of 50, 100, or 200 adjacent SNPs, or one chromosome, or the whole genome. Traditional univariate and bivariate genomic best linear unbiased prediction (GBLUP) models were also run for comparison. Reliabilities were calculated through a resampling strategy and using deterministic formula.

**Results:**

BayesAS models improved prediction reliability for most of the traits compared to GBLUP models and this gain depended on segment size and genetic architecture of the traits. The gain in prediction reliability was especially marked for the protein composition traits β-CN, κ-CN and β-LG, for which prediction reliabilities were improved by 49 percentage points on average using the MT-BayesAS model with a 100-SNP segment size compared to the bivariate GBLUP. Prediction reliabilities were highest with the BayesAS model that uses a 100-SNP segment size. The bivariate versions of our BayesAS models resulted in extra gains of up to 6% in prediction reliability compared to the univariate versions.

**Conclusions:**

Substantial improvement in prediction reliability was possible for most of the traits related to milk protein composition using our novel BayesAS models. Grouping adjacent SNPs into segments provided enhanced information to estimate parameters and allowing the segments to have different (co)variances helped disentangle heterogeneous (co)variances across the genome.

## Background

The protein composition of milk determines its technological characteristics such as the cheese-making properties. Major proteins in milk include the caseins (${\upalpha}$_S1_-, ${\upalpha}$_S2_-, β- and ${{\upkappa}}$-CN) and whey proteins (${\upalpha}$-lactalbumin, and ${{\upbeta}}$-lactoglobulin). The heritability of the relative proportion of these proteins in bovine milk is moderate to high [1–3], which provides the opportunity to alter the protein composition of milk through selective breeding. Prediction of genetic merit for traits related to milk protein composition has never been reported and one reason for this is that measurements of the detailed protein composition of milk is currently limited to experimental samples due to costly and time-consuming analytical techniques.

In livestock breeding, genomic selection has become a successful approach, especially for sex-limited traits, because it speeds up the selection process by reducing generation interval and enables to select new selection candidates at early ages. Accuracy of genomic prediction hinges on a number of factors including size of reference population, heritability of the trait, effective population size, marker density, and the genetic architecture of the trait (in particular, the number of loci that affect the trait and the distribution of their effects) [4–6]. Therefore, prediction accuracy for traits with limited records is still low. However, if the methodology used exploits information about the distribution of the loci that underlie a trait, traits that are controlled by a few quantitative trait loci (QTL) with major effects can be predicted with better accuracy than traits that have a more polygenic nature [7]. Several statistical models have been developed for genomic prediction using genome-wide single-nucleotide polymorphisms (SNPs), which include the Bayesian models (e.g. BayesA and BayesB) of Meuwissen et al. [8], the genomic best linear unbiased prediction (GBLUP) model [9] and several extensions of these models. Compared to GBLUP, the Bayesian variable selection models improve prediction reliability considerably for traits that are controlled by a few QTL with major effects [10, 11]. This is mainly due to the assumption that, in the GBLUP model, the variance does not vary across the genome, i.e., it does not take heterogeneity over segments into account. Unlike GBLUP, Bayesian variable selection models allow the variance of SNP effects to differ among loci [9]. Genome-wide association studies have indicated that a few QTL regions underlie substantial proportions of the genetic variation in traits related to milk protein composition [12]. Hence, it is expected that, for traits related to milk protein composition, a model assuming SNP-specific variances in genomic prediction can result in higher prediction reliability than the GBLUP approach. However when the available dataset is small, as is the case for expensive-to-measure traits, reliable estimation of SNP-specific variances with the Bayesian approach becomes problematic since there are too many parameters to estimate relative to the information available. In such situations, Gianola et al. [13] suggested to group SNPs according to their common variance. Grouping adjacent SNPs might be advantageous for estimating variances reliably by enhancing the amount of information and reducing the number of parameters to estimate. Adjacent SNPs are very likely to be in linkage disequilibrium (LD) with the same QTL and to have the same variance, which allow us to account for heterogeneity between SNP groups. In this context, SNPs must be properly ordered and grouped such that they are realistically in LD with the same QTL while ensuring that their group size is optimum for the reliable estimation of variances.

Another option that is widely used to deal with traits with limited records is to implement multi-trait models, which simultaneously use information from related traits and individuals [14]. In multi-trait analysis, correlation structures between the traits is central to gaining any advantage in prediction reliability over single-trait predictions [15]. Milk protein traits have a low to moderate genetic correlation with routinely recorded traits such as total protein yield [2]. However, while the genome-wide correlation is generally low, specific genomic segments may display high genetic correlations between SNP effects for different traits. Therefore, modeling such heterogeneous covariance patterns may result in improved prediction reliability, when using multi-trait models.

In this study, we report genomic prediction reliabilities for traits related to milk protein composition using a relatively small set of cow data by developing novel Bayesian hierarchical models that account for heterogeneous variance structures across regions of the genome. Furthermore, we extend our novel Bayesian models to bivariate scenarios that model heterogeneous covariance structures between milk protein composition traits measured on cows and a large set of bull data with highly accurate deregressed proofs (DRP) for total protein yield.

## Methods

### Animals and phenotypes

Available data comprised two datasets: a relatively small set of cow data with information on traits related to milk protein composition and a large set of bull data with highly accurate total protein yields from regular milk recordings on daughters. Individuals in the two datasets were genetically related i.e., all the cows had their sires in the bull dataset.

Single morning milk samples were collected once from 650 Danish Holstein cows in 21 herds. Cows were sampled at different stages of lactation (days 9 to 481 in milk) and parity (1 to 4). Liquid chromatography/electrospray ionization–mass spectrometry (LC/ESI–MS) methods were used for profiling milk proteins. Details on the identification and relative quantification of milk proteins are in Jensen et al. [16]. We used these methods to quantify milk proteins, including ${\upalpha}$_S1_-CN, ${\upalpha}$_S2_-CN, ${{\upbeta}}$-CN, ${{\upkappa}}$-CN, ${\upalpha}$-LA, and ${{\upbeta}}$-LG, posttranslational modifications of G-${{\upkappa}}$-CN and ${\upalpha}$_S1_-CN-8P, as well as total protein percentage. In later analyses, ${{\upbeta}}$-CN was excluded from the genetic analysis due to very low estimates of its heritability across models (0.01 to 0.05), which made meaningful predictions difficult to obtain given the small sample size.

DRP for milk protein yield were obtained from 5326 progeny-tested Danish Holstein bulls. Estimated breeding values from the Nordic genetic evaluation in January 2013 were used to derive DRP following the methodology described by Schaeffer [17].

### Genotypes

Genotyping was performed using the BovineHD Illumina Beadchip for 372 cows or the BovineSNP50 Beadchip for the remaining 278 cows and all the bulls. SNPs that overlapped between these two genotyping arrays were combined and subjected to quality control. Quality parameters used to select SNPs were: (1) minimum call rates of 90% for individuals and 95% for loci and (2) exclusion of SNPs with a minor allele frequency (MAF) lower than 5%. Finally, 36,000 SNPs across the 29 bovine autosomes were available for the analyses.

### Models

Hierarchical Bayesian models based on genome segments of different sizes (hereafter collectively called BayesAS models) were developed to predict genomic breeding values (GBV). Univariate and bivariate GBLUP models were used to compare performances of the novel Bayesian models.

#### GBLUP models

Univariate (based on cow data only) and bivariate (based on combined cow data and bull DRP) GBLUP models were implemented using DMU [18]. The general model used for the univariate analysis (ST-GBLUP) was:

1 $$\begin{aligned} y_{ijkl} & = \mu_{i} + parity_{ij} + herd_{ik} + b_{i1} DIM_{l} \\ & \quad + b_{i2} *\exp^{{ - 0.05*DIM_{l} }} +\, g_{il} + e_{1ijkl} , \\ \end{aligned}$$

where *y*_*ijkl*_ are the observations on trait *i* from cow *l*, in parity *j*, and herd *k*; *μ*_*i*_ is the fixed mean effect for trait *i*; *b*_*i*1_ is the regression coefficient for *DIM*_*l*_ in trait *i*, which is a covariate describing the effect of days in milk for each cow *l*; *b*_*i*2_ is the regression coefficient for the Wilmink adjustment ($\exp^{{ - 0.05*DIM_{l} }}$) of days in milk for trait *i*; *e*_1*ijkl*_ is a random residual effect that is assumed to be normally distributed with ${\mathbf{e}}_{1} \sim\, N\left( {0, {\mathbf{I}}_{1} \sigma_{{e_{1} }}^{2} } \right)$, where ${\mathbf{I}}_{1}$ is an identity matrix with dimensions 650 by 650. The effect of *g*_*il*_ is a random additive genetic effect for trait *i* of cow *l* with distribution $N\left( {0, {\mathbf{G}}\sigma_{a}^{2} } \right)$, where ${\mathbf{G}}$ is the genomic relationship matrix between cows with dimension 650 by 650 and *σ*_*a*_^2^ is the genetic variation in trait *i*.

To run a bivariate analysis (MT-GBLUP) of DRP on protein yield and each protein composition trait, DRP were modelled as:

2 $$y_{{DRP_{l} }} = \mu_{DRP} + g_{2l} + e_{2l} ,$$

where $y_{{DRP_{l} }}$ is the DRP for bull *l*; and *μ*_*DRP*_ is the corresponding fixed mean effect. *g*_2*l*_ is the random additive genetic effect for animal *l* for protein yield with distribution $N\left( {0, {\mathbf{G}}_{2} \sigma_{a}^{2} } \right)$, where ${\mathbf{G}}_{2}$ is the genomic relationship matrix for combined cow and bull population with dimension 5976 by 5976. Distribution of the vectors of the two animal effects in the bivariate models are as follows:

$$\begin{aligned}& \left( {\begin{array}{*{20}c} {{\mathbf{g}}_{1} } \\ {{\mathbf{g}}_{2} } \\ \end{array} } \right) \sim\,N\left( {\left( {\begin{array}{*{20}c} 0 \\ 0 \\ \end{array} } \right), \varSigma \otimes {\mathbf{G}}_{2} } \right),\\ & {\text{with}}\;\varSigma = \left( {\begin{array}{*{20}c} {\sigma_{1}^{2} } \\ {\sigma_{1,2} } \\ \end{array} \begin{array}{*{20}c} {\sigma_{1,2} } \\ {\sigma_{2}^{2} } \\ \end{array} } \right) \end{aligned} ,$$

where, in this case, ${\mathbf{g}}_{1}$ is a vector of breeding values for all animals for one of the cow traits based on the covariance matrix ${\mathbf{G}}_{2}$ unlike in Model (1); *σ*_1_^2^ is the genetic variance for each cow trait and *σ*_2_^2^ is the genetic variance for the bull DRP.

The random residual effect *e*_2*l*_, in Model (2), is assumed to be normally distributed with, ${\mathbf{e}}_{2} \sim\, N\left( {0, {\mathbf{I}}_{2} \sigma_{{e_{2} }}^{2} } \right),$ where ${\mathbf{I}}_{2}$ is an identity matrix with dimension 5326 by 5326 and $\sigma_{{e_{2} }}^{2}$ is the residual variation for bull DRP. In the bivariate analysis, the residual covariance for the pair of bivariate traits was set to zero because the observations came from different individuals. The distribution of the vectors of the two residual effects in the bivariate analyses can be described as:

$$\left( {\begin{array}{*{20}c} {{\mathbf{e}}_{1} } \\ {{\mathbf{e}}_{2} } \\ \end{array} } \right) \sim\,N\left( {\left( {\begin{array}{*{20}c} 0 \\ 0 \\ \end{array} } \right), \left( {\begin{array}{*{20}c} {{\mathbf{I}}_{1} \sigma_{{e_{1} }}^{2} } \\ 0 \\ \end{array} \begin{array}{*{20}c} 0 \\ {{\mathbf{I}}_{2} \sigma_{{e_{2} }}^{2} } \\ \end{array} } \right)} \right).$$

The genomic relationship matrix used in the GBLUP models was calculated as described in the first method presented by VanRaden [9].

#### BayesAS models

Models that were proposed initially by Janss [19] were implemented in the MCMC Bayesian framework of the Bayz program (www.bayz.biz). Adjacent SNPs were grouped into non-overlapping genomic segments and the (co)variance for each segment was estimated. Accordingly, six models were implemented, in which a genome segment was defined as: single SNPs or groups of 50, 100, or 200 adjacent SNPs, a complete chromosome or all the SNPs in the genome. The model that considers the whole genome as a segment can be considered basically as a GBLUP model implemented in a Bayesian manner.

Univariate (ST) and bivariate (MT) versions of the BayesAS models were implemented. For the ST-BayesAS models, each protein composition trait (*y*_*ijkl*_) from the cow dataset was run as in the model described below:

3 $$\begin{aligned} y_{ijkl} & = \mu_{i} + parity_{ij} + herd_{ik} \\ & \quad + b_{i1} DIM_{l} + b_{i2} *\exp^{{ - 0.05*DIM_{l} }} + {\mathbf{z}}_{l} {\mathbf{a}}_{i} + e_{1ijkl} . \\ \end{aligned}$$

Model components for fixed effects, covariates and random residual effects are in Model (1). **Z** is a matrix with SNP covariates (centered) with dimensions of the number of individuals (n = 650) by the number of loci (m = 36,000) and ${\mathbf{z}}_{l}$ is a row of genotypes for animal *l*, ${\mathbf{a}}_{i}$ is a vector of SNP effects for trait *i*, with length m and with elements ${\mathbf{a}}_{i} = \left\{ {a_{ijk} } \right\}$, such that *a*_*ijk*_ is the effect of SNP *k* in SNP group *j* for trait *i*.

For the MT-BayesAS models, an additional model component was included to run the DRP on total protein yield from bulls $(y_{{DRP_{l} }} )$ simultaneously with each protein composition trait from cows. The following model was added to run the bivariate MT-BayesAS analyses:

4 $$y_{{DRP_{l} }} = \mu_{DRP} + {\mathbf{z}}_{l} {\mathbf{a}}_{i} + e_{2l} ,$$

**Z** in the MT-BayesAS is a matrix with SNP covariates (centered) with dimensions of the number of individuals (n = 5976) by the number of loci (m = 36,000) and ${\mathbf{z}}_{l}$ is a row of genotypes for animal *l*, ${\mathbf{a}}_{i}$ is a vector of SNP effects for trait *i*, with length m and with elements ${\mathbf{a}}_{i} = \left\{ {a_{ijk} } \right\}$ and the residual term (*e*_2*l*_), is as in Model (2). The index “*i*” here refers to both cow trait and bull DRP run in each bivariate analysis, for sake of simplicity in describing the models. SNP effects between each of the two traits in the bivariate analyses are correlated using latent variables by the following hierarchical model:

5 $$a_{ijk} = r_{0i} *{\mathbf{s}}_{0} + r_{1ij} *{\mathbf{s}}_{1} + a_{ijk}^{*} ,$$

where ${\mathbf{s}}_{0} = \left\{ {s_{0jk} } \right\}$ and ${\mathbf{s}}_{1} = \left\{ {s_{1jk} } \right\}$ are vectors of latent variables with length m, to model average covariance across SNP groups (${\mathbf{s}}_{0}$) and deviations within SNP groups (${\mathbf{s}}_{1}$) using nested regression; $r_{0i}$ is a regression coefficient of ${\mathbf{s}}_{0}$ for all SNPs and $r_{1ij}$ is a regression coefficient of ${\mathbf{s}}_{1}$ for each SNP group *j*; and $a_{ijk}^{*}$ is the residual SNP effect, which is uncorrelated across traits. The latent variables in ${\mathbf{s}}_{0}$ and ${\mathbf{s}}_{1}$ are assumed to be normally distributed with a variance of 1:

$${\mathbf{s}}_{0} \sim\,\, N\left( {0,{\mathbf{I}}} \right)\quad {\text{and}}\quad {\mathbf{s}}_{1} \sim\,N\left( {0,{\mathbf{I}}} \right),$$

where **I** is an identity matrix with dimensions of number of loci (m = 36,000).

Distributional prior assumptions for the regression coefficients of ${\mathbf{s}}_{0}$ and ${\mathbf{s}}_{1}$ are:

$$\begin{aligned} r_{0i} & \sim\,\,U\left( { - \,\infty ,\infty } \right), \\ r_{1ij} & \sim\,\,N\left( {0,\sigma_{{r_{1i} }}^{2} } \right), \\ \sigma_{{r_{1i} }}^{2} & \sim\,\,U\left( {0,\infty } \right), \\ \end{aligned}$$

where *U*() stands for a uniform distribution across the given interval.

The residual SNP effect *a*_ijk_^*^ is assumed to be normally distributed with a mean of 0 and SNP-group specific variance ($\sigma_{{a_{ij}^{*} }}^{2} )$ for which an inverse-Chi square distribution was set with scale *SC*_*i*_^2^ and degrees of freedom *df*_*i*_ for all SNP effects in group *j*:

$$\begin{aligned} a_{ijk}^{*} & \sim\,\,N\left( {0, \sigma_{{a_{ij}^{*} }}^{2} } \right), \\ \sigma_{{a_{ij}^{*} }}^{2} & \sim\,\chi^{ - 2} \left( {SC_{i}^{2} , df_{i} } \right). \\ \end{aligned}$$

The scale parameter *SC*_*i*_^2^ is assumed to have a uniform distribution. The parameter *df*_*i*_ is set so that the spread of the variances of individual SNP-groups around the common scale is controlled (here, a value of 5 was used).

Samples of the posterior distributions of the model parameters are obtained using MCMC techniques, i.e., sampling from conditional distributions. The conditional distributions for all parameters in Eqs. (3), (4) and (5) are normal and for variances are scale-inverse Chi squared. For the parameters ${\mathbf{s}}_{0}$ and ${\mathbf{s}}_{1}$, which are present in the expectation for multiple SNP-effects, the bayz software automatically combines all parts of the likelihoods and combines them with the prior distribution to form the conditional posterior.

${\mathbf{Za}}_{i}$ from Models (3) and (4) computes the genomic values (${\mathbf{g}}_{i} )$ at each MCMC cycle. The total explained genomic variance for trait *i* is computed as the variance of the genomic values from every MCMC cycle:

6 $$\sigma_{i}^{2} = \text{var} \left( {{\mathbf{Za}}_{i} } \right) = \text{var} \left( {{\mathbf{g}}_{i} } \right).$$

The genomic covariance between the cow and bull traits can then be calculated as:

7 $$\sigma_{cow,bull} = \text{cov} \left( {{\mathbf{g}}_{cow} ,{\mathbf{g}}_{bull} } \right),$$

where ${\mathbf{g}}_{cow}$ is a vector of genomic values for all individuals for each cow-trait and ${\mathbf{g}}_{bull}$ is a vector of genomic values of all individuals for total protein yield. Similarly, genetic values for the individuals at SNP group *j* (${\mathbf{g}}_{ij} )$ was calculated at each MCMC sample based on the genotypes and estimated effects of SNPs in group *j* as:

8 $${\mathbf{g}}_{{ij\varvec{ }}} = {\mathbf{Z}}_{j} \varvec{ }{\mathbf{a}}_{ij} ,$$

where ${\mathbf{Z}}_{\varvec{j}}$ is a matrix of covariates for SNPs within group *j*, with size of number of individuals by number of SNPs at group *j*, and ${\mathbf{a}}_{ij}$ is a vector of effects of SNPs at group *j* for trait *i*. Genomic variance for trait *i* at SNP group *j* was then calculated from these MCMC samples of individual genetic values as:

9 $$\sigma_{ij}^{2} = \text{var} \left( {{\mathbf{g}}_{ij} } \right).$$

The proportion of the genomic variance explained by segments was computed for each trait *i* as $\frac{{\sigma_{ij}^{2} }}{{\sigma_{i}^{2} }}$. The genomic covariance for each cow and bull trait at each SNP group *j* was then calculated as:

10 $$\sigma_{cowj,bullj} = \text{cov} \left( {{\mathbf{g}}_{cowj} ,{\mathbf{g}}_{bullj} } \right).$$

Inferences were based on 500,000 Gibbs samples. The first 50,000 samples were discarded as burn-in, and every 500th sample was saved for post-Gibbs analyses. The mean of the variance and covariance terms, which are calculated in each MCMC iteration, is used later. Convergence was assessed using the R package CODA [20].

The BayesAS models presented in this study can be considered as extensions of the Bayes A model of Meuwissen et al. [8], which mainly differ in that estimates of variances are per SNP groups (segments) instead of per single SNP. In this case, taking one SNP as a segment might be considered as an approximation to the BayesA approach. However, there is still a difference in that the scale parameter of the $\chi^{ - 2} \left( {SC_{i}^{2} , df_{i} } \right)$ prior for $\sigma_{{a_{ij}^{*} }}^{2}$ is treated as unknown instead of being fixed. Moreover, the bivariate versions of BayesAS uniquely use latent variables to model covariances between traits.

### Comparison of the predictive ability between models

A resampling strategy using cows in five test sets was implemented to compare models for prediction reliability. Our aim was to avoid sibling relationships between each test set and between the training and test sets. Hence, 197 cows, which had no siblings in the dataset, were selected. In each of the resampled analyses, 100 of the 197 cows were randomly taken for the test set, while the remaining 97 cows from each random sampling were included in the reference population of 550 cows. For all models, prediction reliability for cows was computed as the squared correlation between estimated GBV and the phenotype corrected for fixed effects as in Model (1), divided by heritability estimates [21] from the complete dataset of 650 cows using Model (1). Since the major practical implication of genomic prediction studies is to assess the predictive ability of models for young bulls with no phenotypic record, reliabilities of models in the MT-BayesAS analyses were computed for bulls using standard errors of predicted GBV using the following formula, as described by Mrode [22]:

11 $$1 - \frac{{SEP_{l}^{2} }}{{\sigma_{i}^{2} }},$$

where *SEP*_*l*_ is the standard error of prediction (posterior standard deviations from MCMC samples) of GBV for each bull based on its Gibbs samples for each protein composition trait; and *σ*_*i*_^2^ is the total genomic variance calculated as in Eq. (6), which, as an approximation, was taken as the additive genetic variance. Model reliabilities were computed for all bulls, and the average was taken as the model reliability for the respective trait.

Further analyses were conducted using the Gibbs samples from the 100-SNP segment size MT-BayesAS model to assess prediction reliability when varying the proportion of segments, based on ranking of explained genomic variance. Prediction reliabilities were, accordingly, computed using the top 2% (8), 7% (26), 15% (56), 25% (93), 50% (186), or 75% (279) of all 372 genomic segments included in the analyses. Segments were ranked on estimated variance based on evaluation on the training data with all segments included. Reliabilities were computed for the test sets similarly as in the other BayesAS models and were used to compare the different scenarios.

## Results

### Heritability estimates for milk protein composition traits and genomic correlations with total protein yield

Table 1 presents heritability estimates for traits related to milk protein composition obtained with the ST-GBLUP model, their genome-wide correlations and covariances with total milk protein yield obtained with the MT-GBLUP model. Heritability estimates were high for ${{\upkappa}}$-CN, G-${{\upkappa}}$-CN, ${{\upbeta}}$-LG, and protein percentage. Heritability estimates were moderate for ${\upalpha}$_S2_-CN, but lower for ${\upalpha}$_S1_-CN, ${\upalpha}$_S1_-CN-8P, and ${\upalpha}$-LA. Milk protein composition traits showed very low (− 0.16 to 0.15) genomic correlations with total milk protein yield. Genome-wide correlations with protein yield were negative for ${\upalpha}$_S2_-CN, ${\upalpha}$_S1_-CN-8P, and protein percentage. Standard errors of the correlations were higher than the correlation estimates for all traits except for ${\upalpha}$_S2_-CN and protein percentage.

**Table 1:** Heritability estimates and genome-wide correlations and covariances with total milk protein yield

Trait^a^	h^2^	SE	Covariance	SE	Correlation	SE
${\upalpha}$_S1_-CN	0.14	0.07	0.01	0.05	0.04	0.16
${\upalpha}$_S1_-CN-8P	0.14	0.09	− 0.02	0.05	− 0.07	0.16
${\upalpha}$_S2_-CN	0.33	0.09	− 0.08	0.06	− 0.16	0.12
${{\upkappa}}$-CN	0.69	0.09	0.06	0.05	0.09	0.07
G-${{\upkappa}}$-CN	0.41	0.09	0.0008	0.04	0.0006	0.10
${\upalpha}$-LA	0.15	0.09	0.05	0.05	0.15	0.16
${{\upbeta}}$-LG	0.52	0.10	0.04	0.05	0.07	0.09
Protein %	0.54	0.09	− 0.08	0.06	− 0.14	0.10

Heritability (h^2^) estimates were from the univariate GBLUP analysis; covariances and correlations are from the bivariate GBLUP model

^a^Protein composition expressed as a fraction of the total milk protein percentage by weight wt (wt/wt), protein % expressed as percentage of the total milk yield; individual proteins comprise only the peaks identified as intact proteins and isoforms, i.e., ${\upalpha}$_S1_-CN (comprises ${\upalpha}$_S1_-CN 8P + 9P), ${\upalpha}$_S2_-CN (comprises ${\upalpha}$_S2_-CN 11P + 12P), ${{\upkappa}}$-CN (comprises ${{\upkappa}}$-CN G 1P + unglycosylated ${{\upkappa}}$-CN 1P), where P = phosphorylated serine group. G-${{\upkappa}}$-CN = glycosylated-${{\upkappa}}$-CN; ${\upalpha}$_S1_-CN-8P = ${\upalpha}$_S1_-CN with 8 phosphorylated serine groups

### Prediction reliability of the GBLUP models

Prediction reliabilities were low for all traits (0.03 to 0.21) when using the ST- and MT-GBLUP models (Table 2). Compared to the other protein composition traits, ${{\upbeta}}$-LG (0.21) and ${{\upkappa}}$-CN (0.16) had the highest prediction reliabilities, whereas ${\upalpha}$_S2_-CN and ${\upalpha}$_S1_-CN-8P had the lowest (0.03) when using univariate analysis. There was a slight gain in prediction reliability for ${\upalpha}$_S2_-CN and protein percentage when bivariate analysis was used. There was no improvement in prediction reliability for ${{\upkappa}}$-CN, G-${{\upkappa}}$-CN, ${{\upbeta}}$-LG, or ${\upalpha}$_S1_-CN-8P compared to ST-GBLUP predictions. Prediction reliability was a little lower with the MT-GBLUP model than with univariate prediction for ${\upalpha}$_S1_-CN and ${\upalpha}$-LA.

**Table 2:** Prediction reliability from univariate and bivariate GBLUP models

Trait^a^	ST-GBLUP	MT-GBLUP
${\upalpha}$_S1_-CN	0.11	0.10
${\upalpha}$_S1_-CN-8P	0.03	0.03
${\upalpha}$_S2_-CN	0.03	0.06
${{\upkappa}}$-CN	0.16	0.16
G-${{\upkappa}}$-CN	0.14	0.14
${\upalpha}$-LA	0.12	0.11
${{\upbeta}}$-LG	0.21	0.21
Protein %	0.10	0.12

^a^Protein composition expressed as a fraction of the total milk protein percentage by weight wt (wt/wt), protein % expressed as percentage of the total milk yield; individual proteins comprise only the peaks identified as intact proteins and isoforms, i.e., ${\upalpha}$_S1_-CN (comprises ${\upalpha}$_S1_-CN 8P + 9P), ${\upalpha}$_S2_-CN (comprises ${\upalpha}$_S2_-CN 11P + 12P), ${{\upkappa}}$-CN (comprises ${{\upkappa}}$-CN G 1P + unglycosylated ${{\upkappa}}$-CN 1P), where P = phosphorylated serine group. G-${{\upkappa}}$-CN = glycosylated-${{\upkappa}}$-CN; ${\upalpha}$_S1_-CN-8P = ${\upalpha}$_S1_-CN with 8 phosphorylated serine groups

### Genome segment-wise variances for milk protein composition traits and covariance with total protein yield

**Fig. 1:**
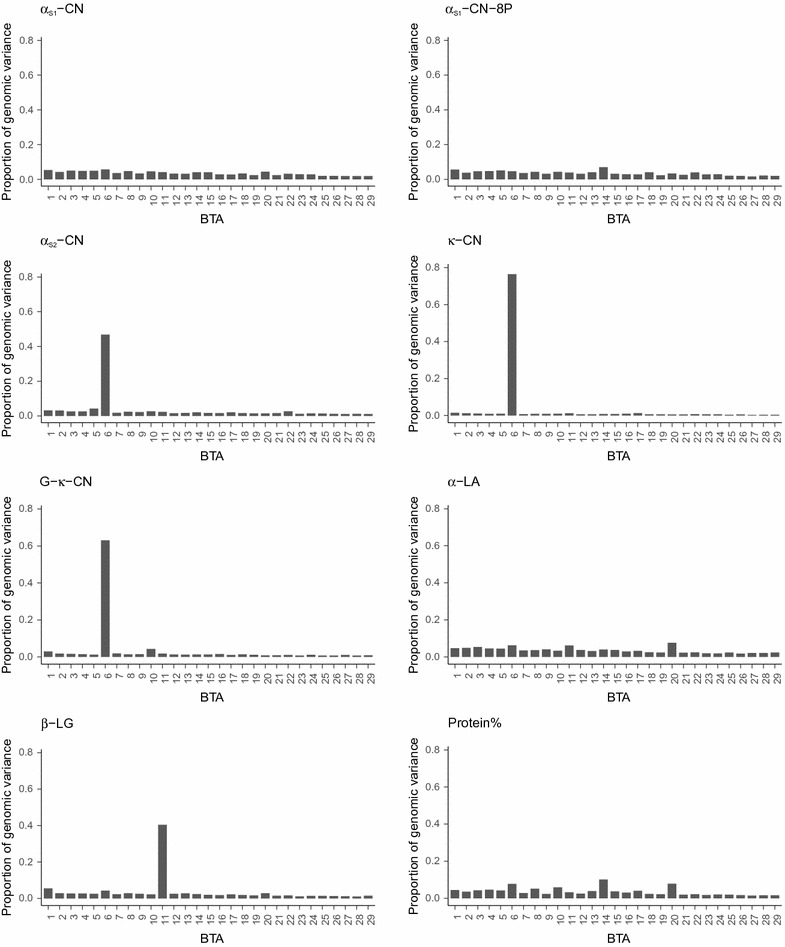
Proportion of genomic variance explained by each chromosome. Proportion of the genomic variance in the milk protein composition traits explained by each chromosome from the ST-BayesAS model taking chromosomes as segments

Figure 1 presents the proportion of genomic variance in milk composition traits explained by each chromosome using the ST-BayesAS model. Marked differences were observed in the proportion of genomic variance explained by genome segments across the traits. For some of the protein composition traits, a single chromosome explained up to or more than half of the genomic variance. For instance, *Bos taurus* (BTA) chromosome 6 explained 76, 63 and 47% of the genomic variance for ${{\upkappa}}$-CN, G-${{\upkappa}}$-CN and ${\upalpha}$_S2_-CN, respectively. Likewise, 40% of the genomic variance for ${{\upbeta}}$-LG was explained by BTA11 alone.

**Fig. 2:**
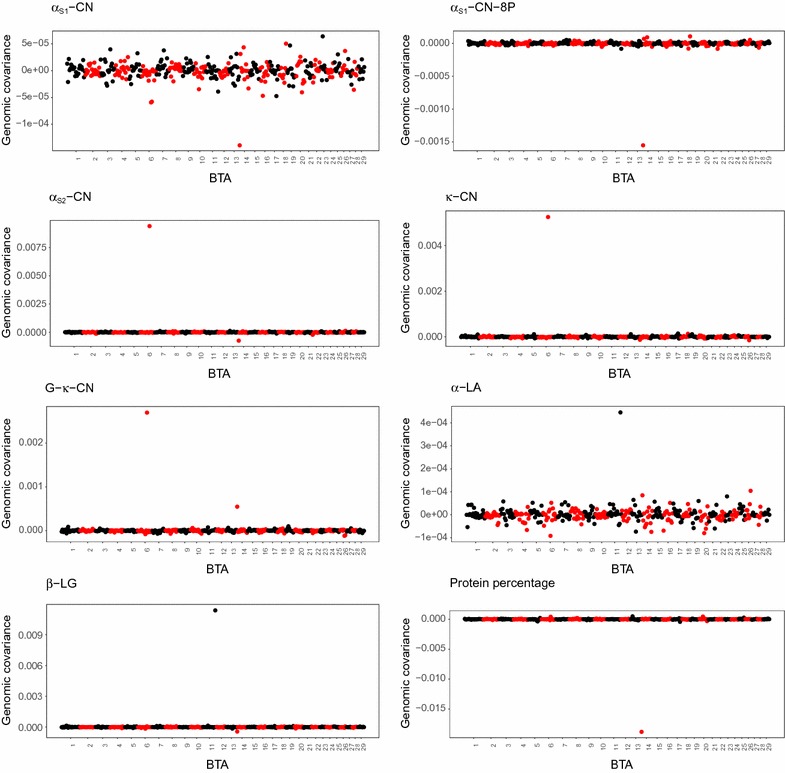
Covariance between each protein composition trait with total protein yield explained by 100-SNP genomic segments

Figure 2 shows the covariances between traits related to milk protein composition and total protein yield explained by genomic segments of 100 SNPs. Across the traits, some segments explained a large part of the covariance, whereas others accounted for nearly no covariance. Covariances between total milk protein yield and a particular trait were positive for some segments and negative for others. For G-${{\upkappa}}$-CN, ${{\upkappa}}$-CN, ${{\upbeta}}$-LG, ${\upalpha}$_S2_-CN, and protein percentage, a few segments showed peaks for the explained covariance. Segment 106, corresponding to a group of 100 adjacent SNPs on BTA6, explained a large amount of positive covariance of ${\upalpha}$_S2_-CN, ${{\upkappa}}$-CN, and G-${{\upkappa}}$-CN with total protein yield. Similarly, a sizable proportion of the covariance between ${{\upbeta}}$-LG and protein yield was explained by a single segment on BTA11. A segment on BTA14 explained a substantial part of the negative covariance between protein percentage and protein yield. The same segment showed a peak for the covariance between ${\upalpha}$_S1_-CN-8P and total milk protein yield compared to the rest of the segments. Although some segments explained relatively more covariance between ${\upalpha}$_S1_-CN and total protein yield and between ${\upalpha}$-LA and total protein yield compared to other segments, the actual covariance values explained by these segments were very low (note the difference in y-axis scales between plots in Fig. 2).

### Prediction reliability with BayesAS models

**Fig. 3:**
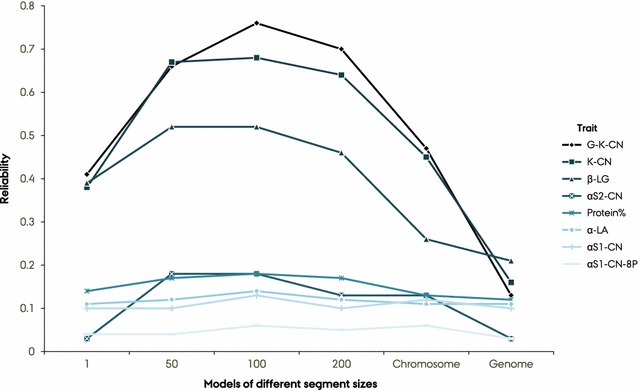
Prediction reliability across MT-BayesAS models. Reliability of models according to segment sizes of 1, 50, 100, and 200 SNPs, chromosome, and whole genome. G-${{\upkappa}}$-CN = glycosylated-${{\upkappa}}$-CN; ${\upalpha}$_S1_-CN-8P = ${\upalpha}$_S1_-CN with eight phosphorylated serine groups

Prediction reliabilities for cows using the BayesAS models were generally high compared to those obtained with the GBLUP models across all traits. Prediction reliabilities using both the MT- (Fig. 3) and ST-BayesAS models were generally high for most of the highly heritable traits, such as ${{\upkappa}}$-CN, G-${{\upkappa}}$-CN, and ${{\upbeta}}$-LG, using different segment sizes. Using the 100-SNP segment size resulted in the highest prediction reliability for all studied protein composition traits in both univariate and bivariate versions of the BayesAS models. Prediction reliabilities using the 100-SNP segment size with the MT-BayesAS model were 0.76 for G-${{\upkappa}}$-CN, 0.68 for ${{\upkappa}}$-CN, and 0.52 for ${{\upbeta}}$-LG. Expanding the segment size to include all SNPs on a chromosome or the whole genome resulted in the lowest prediction reliabilities with the BayesAS models. The performance of the whole-genome-based model was similar to that of the respective GBLUP models. With the MT-BayesAS model, improvement in prediction reliability reached 63% for G-${{\upkappa}}$-CN, 52% for ${{\upkappa}}$-CN, 31% for ${{\upbeta}}$-LG, and 15% for ${\upalpha}$_S2_-CN when using the 100-SNP-based model compared to the whole-genome-based model. Prediction reliabilities were low for ${\upalpha}$_S1_-CN, ${\upalpha}$-LA, and ${\upalpha}$_S1_-CN-8P for all BayesAS models and improved minimally by using the 100-SNP-based model compared to the whole-genome approach. The 50- and 100-SNP models performed similarly well for ${{\upbeta}}$-LG. However, for the other proteins, the 100-SNP model outperformed both the 50- and 200-SNP based models, which generally showed comparable results. Using the single-SNP segment size resulted in lower performance compared to the 50-, 100-, and 200-SNP-based models for all traits. Prediction reliabilities computed for ${{\upbeta}}$-LG and protein percentage using the single-SNP-based MT-BayesAS model were better than when each chromosome (by 13 and 1 percentage points) or the whole genome was used as the segment (by 18 and 2 percentages points), respectively.

In general, slight additional gains in prediction reliability were achieved using the MT-BayesAS models compared to the univariate BayesAS model (Table 3), i.e., 6 and 5 percentage points for G-${{\upkappa}}$-CN and ${{\upkappa}}$-CN using 100 SNP-segments and the average improvement with this segment size was 3 percentage points. However, improvement in prediction reliability from the MT-BayesAS models declined when the whole genome was taken as segment, which resulted basically in similar performances than the ST version except for ${{\upbeta}}$-LG.

**Table 3:** Prediction reliability from univariate and bivariate BayesAS models

Trait^a^	BayesAS-1SNP		BayesAS-100SNP		BayesAS-Genome	
	MT	ST	MT	ST	MT	ST
${\upalpha}$_S1_-CN	0.10	0.09	0.13	0.09	0.10	0.09
${\upalpha}$_S1_-CN-8P	0.04	0.02	0.06	0.03	0.03	0.03
${\upalpha}$_S2_-CN	0.03	0.03	0.18	0.16	0.03	0.03
${{\upkappa}}$-CN	0.38	0.37	0.68	0.63	0.16	0.16
G-${{\upkappa}}$-CN	0.41	0.39	0.76	0.70	0.13	0.14
${\upalpha}$-LA	0.11	0.09	0.14	0.14	0.11	0.11
${{\upbeta}}$-LG	0.39	0.39	0.52	0.50	0.21	0.19
Protein %	0.14	0.14	0.18	0.17	0.12	0.11

^a^Protein composition expressed as a fraction of the total milk protein percentage by weight wt (wt/wt), protein % expressed as percentage of the total milk yield; individual proteins comprise only the peaks identified as intact proteins and isoforms,i.e., ${\upalpha}$_S1_-CN (comprises ${\upalpha}$_S1_-CN 8P + 9P), ${\upalpha}$_S2_-CN (comprises ${\upalpha}$_S2_-CN 11P + 12P), ${{\upkappa}}$-CN (comprises ${{\upkappa}}$-CN G 1P + unglycosylated ${{\upkappa}}$-CN 1P), where P = phosphorylated serine group. G-${{\upkappa}}$-CN = glycosylated-${{\upkappa}}$-CN; ${\upalpha}$_S1_-CN-8P = ${\upalpha}$_S1_-CN with 8 phosphorylated serine groups

### Reliabilities of models for bulls

Table 4 shows the reliabilities of the MT-BayesAS models for bulls with segments of different sizes. Prediction reliability computed for the cow datasets was higher than that for bulls for G-${{\upkappa}}$-CN while the reverse was found for ${\upalpha}$_S2_-CN. Higher model reliabilities were computed for bulls for ${\upalpha}$_S2_-CN, ${{\upkappa}}$-CN, G-${{\upkappa}}$-CN and ${{\upbeta}}$-LG with the 50- and 100-SNP segments compared to the other MT-BayesAS models. On the contrary, prediction reliability did not vary much across models for ${\upalpha}$_S1_-CN, ${\upalpha}$-LA, ${\upalpha}$_S1_-CN-8P and protein percentage, which had relatively low reliabilities. Prediction reliabilities obtained from the MT-GBLUP model were similar to those from the genome-based MT-BayesAS model for all traits and hence are not presented in Table 4.

**Table 4:** Model reliability for bulls across the MT-BayesAS models

Trait^a^	MT-BayesAS model reliability					
	1	50	100	200	Chromosome	Genome
${\upalpha}$_S1_-CN	0.05	0.06	0.04	0.06	0.05	0.06
${\upalpha}$_S1_-CN-8P	0.06	0.06	0.06	0.06	0.06	0.07
${\upalpha}$_S2_-CN	0.12	0.32	0.32	0.26	0.21	0.14
${{\upkappa}}$-CN	0.56	0.71	0.71	0.68	0.56	0.21
G-${{\upkappa}}$-CN	0.42	0.56	0.56	0.54	0.39	0.15
${\upalpha}$-LA	0.07	0.07	0.08	0.08	0.08	0.06
${{\upbeta}}$-LG	0.37	0.50	0.51	0.49	0.27	0.19
Protein %	0.23	0.22	0.22	0.21	0.19	0.18

^a^Protein composition expressed as a fraction of the total milk protein percentage by weight wt (wt/wt), protein % expressed as percentage of the total milk yield; individual proteins comprise only the peaks identified as intact proteins and isoforms,i.e., ${\upalpha}$_S1_-CN (comprises ${\upalpha}$_S1_-CN 8P + 9P), ${\upalpha}$_S2_-CN (comprises ${\upalpha}$_S2_-CN 11P + 12P), ${{\upkappa}}$-CN (comprises ${{\upkappa}}$-CN G 1P + unglycosylated ${{\upkappa}}$-CN 1P), where P = phosphorylated serine group. G-${{\upkappa}}$-CN = glycosylated-${{\upkappa}}$-CN; ${\upalpha}$_S1_-CN-8P = ${\upalpha}$_S1_-CN with 8 phosphorylated serine groups

### Prediction reliabilities with selected genome segments

**Fig. 4:**
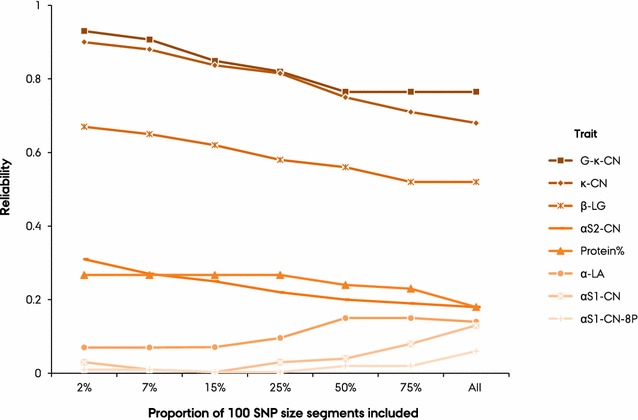
Reliability of prediction using various proportions of genomic segments. Predictions were based on post-Gibbs analyses of samples from the MT-100-BayesA model. Segments were ranked based on explained covariance separately for each training set

Figure 4 shows prediction reliabilities according to the proportion of selected 100-SNP segments used in the prediction. Using fewer segments that explain large proportions of the variances resulted in higher predictive ability for G-${{\upkappa}}$-CN, ${{\upkappa}}$-CN, ${{\upbeta}}$-LG, ${\upalpha}$_S2_-CN, and protein percentage. For these traits, prediction reliability using only 2% (8) of the top-ranked segments resulted in the highest reliability, whereas prediction reliability decreased as more segments were added. In contrast, prediction reliability increased as more segments were added for ${\upalpha}$-LA, ${\upalpha}$_S1_-CN, and ${\upalpha}$_S2_-CN-8P, with the highest reliability obtained when all segments were used for prediction.

## Discussion

### ST-GBLUP versus MT-GBLUP models

Using only the cow dataset with the GBLUP model resulted in low prediction reliability, due to the small size of the training dataset. Reference population size is a key factor that affects reliability of genomic prediction in cattle [4, 7, 23]. Moreover, a small sample size may not sufficiently reflect the genetic variability. For instance, considering a subset of the cow dataset used in this study, Poulsen et al. [24] showed that the genetic variation of the *CSN1N1* gene was very low in Danish Holstein, with most individuals having the *BB* genotype, which may explain the lower prediction reliability for ${\upalpha}$_S1_-CN and its sub-fraction ${\upalpha}$_S1_-CN-8P.

Although information on total protein yield from a large number of bulls was added when using the MT-GBLUP model, prediction reliabilities were as poor as, or even worse than, those in the univariate analysis. Thus, addition of information from total milk protein yield was not sufficient to offset the computational burdens of the bivariate analyses, due to the low genome-wide correlation between protein yield and composition traits. Among the milk proteins, the highest genome-wide correlation with total protein yield was measured for ${\upalpha}$_S2_-CN (− 0.16) and protein percentage (− 0.14), for which the MT-GBLUP model resulted in slightly improved prediction reliabilities for cows and bulls. Although ${\upalpha}$-LA had a correlation of 0.15 with total protein yield, the standard error of the correlation was higher than the correlation estimate (0.16). Although the data used was limited, our findings on genome-wide correlations were comparable to results from previous studies. In the literature, genetic correlations between milk protein percentage and protein yield in different dairy cattle breeds are low, in general [25–27].

Moreover, all the bivariate analyses in our study involved combination of data on different scales, which may have influenced the computed reliabilities. DRP for milk protein yield were expressed on a lactation basis (305-day production), whereas protein composition traits and percentage were related to one morning milk sample. In our study, prediction reliabilities for the traits related to milk protein composition traits were expected to improve if both traits in the bivariate analyses were on a similar scale.

### Predictive ability of BayesAS models

Prediction reliabilities from the resampling showed large improvements with the ST- and MT-BayesAS models compared to their GBLUP counterparts. The BayesAS models allow for different variances and covariances by SNP groups, which can deviate from the genome-wide (co)variance. This was especially important for some traits for which one or two key segments alone explained a large part of the total variance. Grouping adjacent SNPs seems to have helped to obtain more reliable estimates from a small dataset while allowing the segments to have different variances that disentangled heterogeneous (co)variance patterns and improved prediction reliability. Similarly, a simulation study by Shariati et al. [28] showed that prediction reliability based on SNP grouping was better than that obtained by SNP-BLUP methods. SNP grouping in the study of Shariati et al. [28] was based on similar effect sizes. Other grouping options also exist, e.g. depending on LD between SNPs [29]. The BayesAS models can also be used to implement such grouping strategies for which segment sizes might vary depending on LD or effect size similarity.

Prediction reliability with the BayesAS models appears to depend highly on the segment sizes considered and the genetic architecture of the traits. Comparison between the BayesAS models with different segment sizes showed that grouping 100 adjacent SNPs resulted in superior performance for all proteins. Grouping 50 SNPs was as predictive as the models based on 100-SNP segments for all traits except G-${{\upkappa}}$-CN for which prediction reliability improved by 9 percentage points with the 100-SNP segment size model. Taking each SNP as a segment resulted in lower prediction reliability than groups of 50, 100, or 200 adjacent SNPs for most traits. With our BayesAS models, prediction reliabilities decreased as segment size increased beyond 100 SNPs in both the univariate and bivariate analyses. The lowest reliabilities were obtained when considering each chromosome or the whole genome as segments. In other words, the (co)variance between segments was diluted as segment size increased beyond 100 SNPs. Similarly, Brøndum et al. [30] reported that using a segment size of 100 SNPs resulted in the highest accuracy in an across-breed genomic prediction study for protein, fat, and milk yield using 465,000 SNPs. Defining the optimal segment size, in terms of number of adjacent SNPs, is critical to achieving meaningful gains from the novel models presented here. Optimal segment size should be established for each specific situation, for instance through some resampling strategy, considering the SNP array, species, and LD in the population.

The gain in prediction reliability from using different segment sizes in the BayesAS models also varied across the traits. In both the ST- and MT-BayesAS models, differences in prediction reliability between segment sizes were very large for G-${{\upkappa}}$-CN, ${{\upkappa}}$-CN, ${\upalpha}$_S2_-CN, and ${{\upbeta}}$-LG, whereas across all models they were smaller for ${\upalpha}$_S1_-CN, ${\upalpha}$_S1_-CN-8P, or ${\upalpha}$-LA. These results are likely related to the genetic architecture of the protein composition traits investigated. Previous genome-wide association studies found that the proportions of ${{\upkappa}}$-CN, ${\upalpha}$_S2_-CN, and ${{\upbeta}}$-LG in milk are controlled by major QTL on BTA6 and 11 [12], which carry the casein gene cluster and the gene encoding ${{\upbeta}}$-LG [31], respectively. On the one hand, a single chromosome could explain a very large proportion of the variance for some protein composition traits, including G-${{\upkappa}}$-CN, ${{\upkappa}}$-CN, ${{\upbeta}}$-LG, and ${\upalpha}$_S2_-CN, which showed the largest improvement in reliability when the heterogeneity of variances across the genome segments was accounted for. On the other hand, the proportion of explained variance by each chromosome was very small for ${\upalpha}$_S1_-CN and ${\upalpha}$-LA, which indicates that many segments contribute small proportions to the average variance. Similarly, Buitenhuis et al. [31] found no major region that was significantly associated with ${\upalpha}$_S1_-CN in the Danish Holstein population, which could be associated to the low genetic variability of the *CSN1N1* gene reported for this population by Poulsen et al. [24]. This result indicates that SNP grouping is more useful for traits that are controlled by QTL with major effects.

Comparison between the univariate and bivariate versions of our BayesAS models showed that for the most informative traits, the MT version resulted in further improvements in prediction reliability of up to 6 percentage points for segment sizes of 100 and 50. While further improvements in prediction reliability of up to 6% from the MT-BayesAS over the univariate versions are still important, it was generally lower than expected. Further investigations are required to understand the impact of genetic architecture of the indicator trait(s) on the potential advantages, over univariate analysis, of our bivariate BayesAS models.

A few segments explained a substantial proportion of the genomic variance for traits related to milk protein composition and their covariance with protein yield. Thus, we investigated the reliability of predictions based on only a few of the best-explaining 100-SNP segments. Predictions based on only 2% (8/372) of the genome segments resulted in the highest prediction reliability for G-${{\upkappa}}$-CN, ${{\upkappa}}$-CN, ${{\upbeta}}$-LG, and ${\upalpha}$_S2_-CN. For these proteins, prediction reliability decreased as more segments were added. Inclusion of more segments that explained a smaller proportion of the (co)variance added noise rather than meaningful information. Similarly, in a simulation study based on a GBLUP approach, Sarup et al. [32] demonstrated that including non-causal markers led to dilution of the effect of causal markers and reduced predictive ability. For other protein composition traits, including ${\upalpha}$_S1_-CN-8P, ${\upalpha}$_S1_-CN, and ${\upalpha}$-LA, prediction reliability improved as more segments were included, with the highest prediction reliability being obtained when all segments were considered. This result is in agreement with our finding on the proportions of genomic covariance explained by 100-SNP segments, where many segments across the genome contributed small proportions of the average covariance between these traits and total protein yield. In this study, we have used the same dataset to rank the top segments and do the prediction. This could lead to overestimation of reliability and introduce prediction bias. However, such bias is expected to be minimal as the SNP effects in these top segments are re-estimated for prediction with the different proportion of segments.

## Conclusions

A novel BayesAS model, which allows exploring and modeling heterogeneous variance and covariance patterns across genomic regions, improved prediction reliabilities for milk protein composition traits with a small dataset compared to the GBLUP and single-SNP based Bayesian models. The number of adjacent SNPs grouped together affected prediction reliability for the BayesAS models. A segment size of 100 SNPs gave the highest prediction reliability using 36,000 SNPs spread across the genome. For the most informative traits (highest genomic reliability), a further gain in reliability was observed when using the bivariate versions of our BayesAS models compared to univariate counterparts. Our results also show that the gains in prediction reliability achieved by SNP grouping depend on the genetic architecture of the traits. A future study with simulated data would be useful to test our novel BayesAS models with larger datasets.
